# Alkyne hydroarylation with Au N-heterocyclic carbene catalysts

**DOI:** 10.3762/bjoc.9.29

**Published:** 2013-02-05

**Authors:** Cristina Tubaro, Marco Baron, Andrea Biffis, Marino Basato

**Affiliations:** 1 Dipartimento di Scienze Chimiche, Università degli Studi di Padova, via Marzolo 1, 35131 Padova, Italy

**Keywords:** alkyne, C–H functionalization, cyclisation, gold, hydroarylation, N-heterocyclic carbenes

## Abstract

Mono- and dinuclear gold complexes with N-heterocyclic carbene (NHC) ligands have been employed as catalysts in the intermolecular hydroarylation of alkynes with simple unfunctionalised arenes. Both mono- and dinuclear gold(III) complexes were able to catalyze the reaction; however, the best results were obtained with the mononuclear gold(I) complex IPrAuCl. This complex, activated with one equivalent of silver tetrafluoroborate, exhibited under acidic conditions at room temperature much higher catalytic activity and selectivity compared to more commonly employed palladium(II) catalysts. Moreover, the complex was active, albeit to a minor extent, even under neutral conditions, and exhibited lower activity but higher selectivity compared to the previously published complex AuCl(PPh_3_). Preliminary results on intramolecular hydroarylations using this catalytic system indicate, however, that alkyne hydration by traces of water may become a serious competing reaction.

## Introduction

**Scheme 1:**
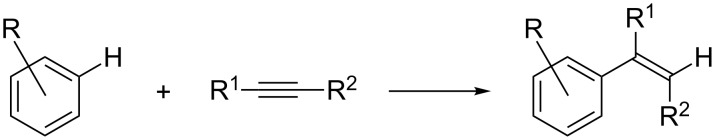
Hydroarylation of alkynes.

The hydroarylation of alkynes (Scheme 1) is arguably one of the most intensively studied reactions leading to aromatic C–H bond functionalization [1–7]. In this reaction, the C–H bond of an arene adds formally *trans* to the triple bond of an alkyne, generally forming the thermodynamically less favoured *cis*-arylalkene as the major product.

The study of this reaction was pioneered by the group of Fujiwara (hence the alternative name “Fujiwara reaction” for the intermolecular hydroarylation of alkynes) using mainly palladium(II) salts as catalyst [8–10]. Palladium complexes with N-heterocyclic carbene (NHC) ligands have since been showcased as highly efficient catalysts for this reaction [11–14]. Alternative catalytic systems based on salts or complexes of other noble metals, such as platinum [15–17], gold [18–19], or rhodium [20], as well as of non-noble, electrophilic metals [21–26] have also been successfully employed. Finally, an even greater number of catalysts have been proposed over the years to promote alkyne hydroarylation in an intramolecular fashion [1–7]. In such reactions, often simply termed cyclisation reactions, the arene and the alkyne are linked through a tether, the nature of which can range from simple alkyl groups to ether, amino, ester or amido groups; depending on the nature and length of the tether, different kinds of unsaturated poly(hetero)cyclic compounds can be conveniently synthesized.

Recently, the unique ability of gold centres to activate C–C triple bonds towards nucleophilic attack has clearly emerged in the literature [27–34]. In the light of the above, it is surprising that the number of studies on the use of gold species as catalysts for alkyne hydroarylation is still quite limited. A substantial number of reports on the intramolecular cyclisation of arenes with tethered alkyne moieties using gold(I) or, to a lesser extent, gold(III) catalysts can be found in the literature [35–46]; however, only one additional example, beyond the two early reports by Reetz and Sommer [18] and by Shi and He [19], of gold-catalysed intermolecular hydroarylation has been described, albeit concerning 2-substituted oxazoles as the reaction partner [47]. Investigations on the intramolecular variant have focused mainly on the nature of the aromatic moiety that adds to the alkyne, and on the nature and/or length of the tether, whereas concerning the alkyne moiety, only terminal, electron-rich alkyne groups (propargylic moieties in most instances) were employed, with very few exceptions [36,40,44]. Finally, concerning the nature of the employed catalysts, simple gold salts or phosphino complexes of gold(I) were utilized in the majority of cases, although in recent years an increasing number of studies have been dealing with the application of NHC complexes of gold for these and related reactions [48–53].

We have an ongoing interest in the development of novel catalysts for the hydroarylation of alkynes and have extensively investigated the ability of palladium(II) complexes with chelating N-heterocyclic dicarbene ligands to promote this reaction [12–14]. Recently, we have extended our interest in the organometallic chemistry of such ligands to group 11 metals, in particular gold(I) and gold(III) centers [54–56]. In the present contribution we would like to assess the catalytic efficiency of such gold complexes with NHC ligands for the hydroarylation of alkynes.

## Results and Discussion

**Figure 1:**
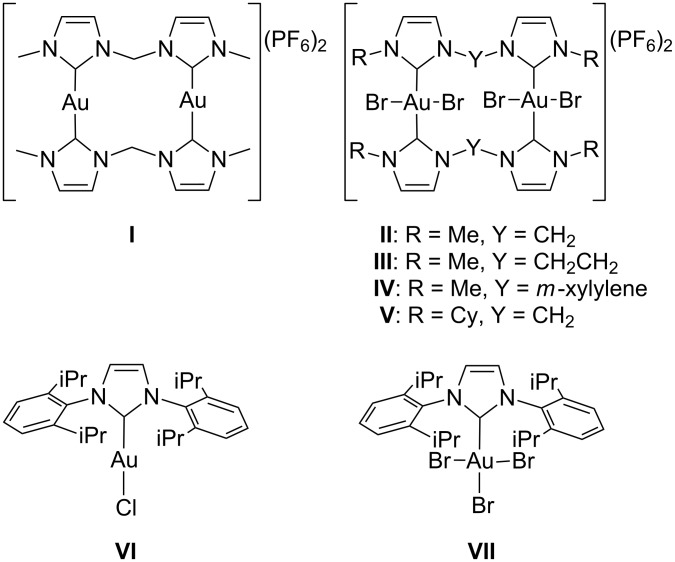
Gold(I) and gold(III) NHC complexes employed as catalysts in this study.

We recently reported on the synthesis of dinuclear gold(I) complexes with bridging dicarbene ligands [55], as well as on the preparation of the corresponding dinuclear gold(III) analogues, which are obtained from the former upon oxidation with bromine [54]. Complexes **I**–**V** were now tested as catalysts in standard intermolecular hydroarylation reactions, together with two mononuclear gold complexes previously reported in the literature, namely complexes **VI** (also termed IPrAuCl) [57] and **VII** (also termed IPrAuBr_3_) [58] (Figure 1).

**Scheme 2:**
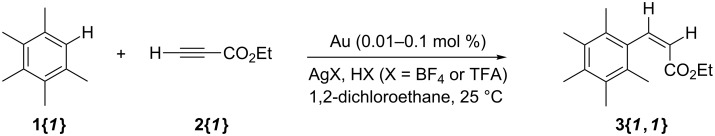
Hydroarylation of ethyl propiolate with pentamethylbenzene.

The standard reaction between pentamethylbenzene and ethyl propiolate (Scheme 2) was initially taken as the benchmark for catalyst evaluation. Initial attempts were performed at room temperature (25 °C) with very low levels of complex **II** as catalyst (0.005 mol %) by using trifluoroacetic acid (HTFA) or HBF_4_ as acidic medium plus 0.02 mol % AgTFA or AgBF_4_, respectively, as co-catalyst to remove bromides from the coordination sphere of the gold centres, thereby liberating coordination sites at the metal and boosting its electrophilicity.

**Figure 2:**
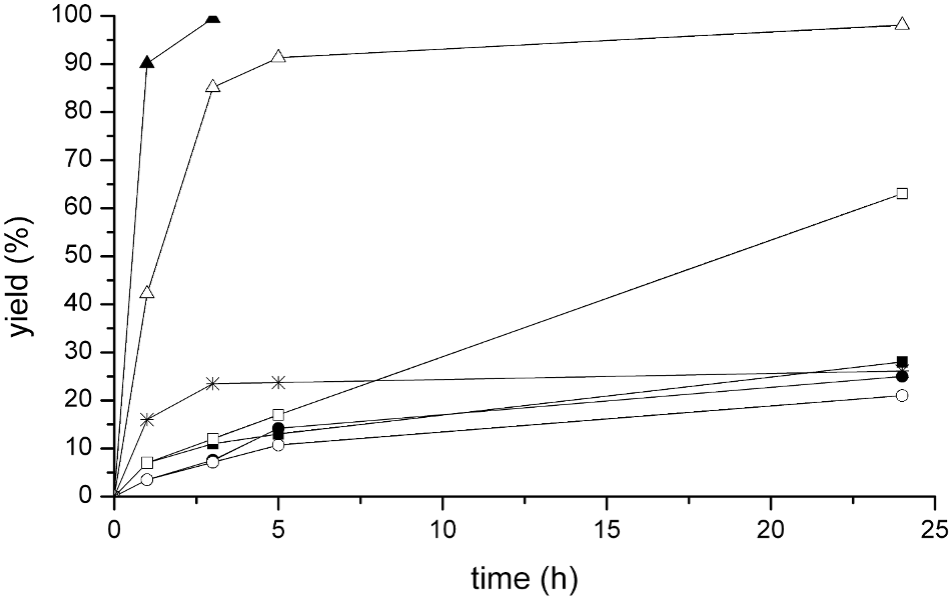
Yield in **3{*1*,*1*}** versus time diagram for the reaction of pentamethylbenzene and ethyl propiolate catalysed by complexes **II**–**VII** and KAuBr_4_ at room temperature in HBF_4_ and with added AgBF_4_: complex **II** (black squares); complex **III** (circles); complex **IV** (squares); complex **V** (black circles); complex **VI** (black triangles); complex **VII** (triangles); KAuBr_4_ (asterisks). Reaction conditions: 1 equiv arene, 1 equiv alkyne, 1 equiv tetrafluoroboric acid, 0.1 mol % Au, 0.1–0.4 mol % AgBF_4_, 1,2-dichloroethane, 25 °C.

The obtained results established that the complex was inactive when HTFA was employed as the acidic medium, whereas 20% yield of the desired product **3{*1*,*1*}** was obtained after 18 h with HBF_4_. Consequently, a screening of the catalytic efficiency of the various complexes was carried out with the latter acidic medium; the amount of catalyst was increased tenfold (to 0.1 mol % Au) in order to achieve faster reaction rates, whereas the amount of employed AgBF_4_ co-catalyst was always stoichiometrically equivalent to the amount of bromide in the employed complex. It should be mentioned that under these reaction conditions neither AgBF_4_ nor HBF_4_ promote the reaction, as previously demonstrated by us in investigations on related palladium(II) catalysts for the same reaction [13]. The conversion curves obtained with the various catalysts are reported in Figure 2.

As expected, the dinuclear dicarbene gold(I) complex **I** was found to be inactive for the reaction, as the NHC ligands saturate the coordination sphere of the gold(I) centres. On the other hand, the dinuclear dicarbene gold(III) complexes **II**–**V**, for which 4 equiv of AgBF_4_ with respect to the complex were added, turned out to be active. All dinuclear complexes exhibited very similar initial activity in the first few hours of reaction. This observation indicates that the reactivity of the complexes is not hampered by steric effects, as complexes with ligands of widely different steric bulk, such as **II** and **V**, exhibit similar performance. On the other hand, the complexes deactivate with time at different rates, depending on the nature of the employed dicarbene ligand. Complex **IV** turned out to be the catalyst most resistant to deactivation.

When catalysts **VI**, **VII** and KAuBr_4_ were employed together with the corresponding amount of AgBF_4_ co-catalyst, higher initial activities compared to the dinuclear dicarbene gold(III) catalysts were recorded. However, whereas KAuBr_4_ was very quickly and completely deactivated, catalysts **VI** and **VII** retained their activity, highlighting the importance of the NHC ligand in stabilizing the catalytically active species. Catalyst **VI** (IPrAuCl) was particularly efficient and able to effect over 90% yield in just 1 h with complete selectivity for the hydroarylation product. Remarkably, compared to the palladium(II) complexes with chelating N-heterocyclic dicarbene ligands previously investigated by us as catalysts for the same reaction under identical reaction conditions [13], complex **VI** exhibits higher catalytic activity and complete selectivity for the insertion of only one alkyne molecule into the aromatic C–H bond, whereas the palladium(II) complexes predominantly yielded the product deriving from the insertion of two alkyne molecules. The catalytic efficiency of complexes **VI** and **VII** was evaluated with other arene and alkyne substrates under the same reaction conditions and the results are reported in Table 1.

**Table 1:** Hydroarylation of alkynes using gold NHC catalysts: screening of different arenes and alkynes.^a^

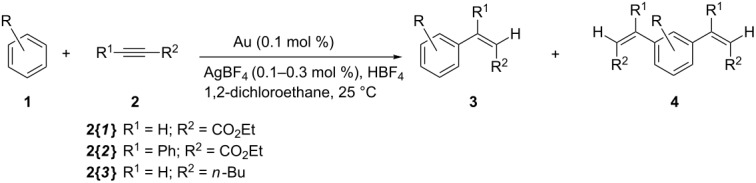								

Catalyst	Arene	Alkyne	Time (h)	Arene conversion, % (alkyne conversion)	Yield (%)^b^			

**VI**	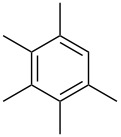 **1{*1*}**	**2{*1*}**	1 3	90 (90) >99 (>99)	**3{*1*,*1*}**	90 >99		
**VI**	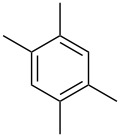 **1{*2*}**	**2{*1*}**	1 5	68 (94) 72 (100)	**3{*2*,*1*}**	42 44	**4{*2*,*1*}**	26 28
**VI**	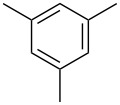 **1{*3*}**	**2{*1*}**	1 5	72 (95) 74 (98)	**3{*3*,*1*}**	49 50	**4{*3*,*1*}**	23 24
**VI**	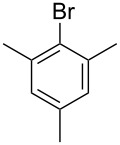 **1{*4*}**	**2{*1*}**	5	35 (38)	**3{*4*,*1*}**	32	**4{*4*,*1*}**	3
**VI**	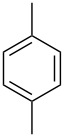 **1{*5*}**	**2{*1*}**	1 5	20 (20) 45 (45)	**3{*5*,*1*}**	20 45	**4{*5*,*1*}**	0 0
**VII**	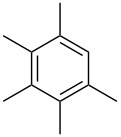 **1{*1*}**	**2{*1*}**	1 5	43 (43) 91 (91)	**3{*1*,*1*}**	43 91		
**VII**	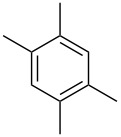 **1{*2*}**	**2{*1*}**	1 5	18 (19) 58 (72)	**3{*2*,*1*}**	17 45	**4{*2*,*1*}**	1 13
**VII**	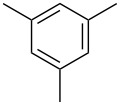 **1{*3*}**	**2{*1*}**	1 5	66 (86) 71 (93)	**3{*3*,*1*}**	46 50	**4{*3*,*1*}**	20 22
**VI**	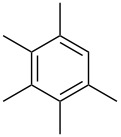 **1{*1*}**	**2{*2*}**	1 5	23 (23) 58 (58)	**3{*1*,*2*}**	23 58		
**VI**	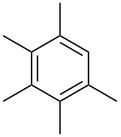 **1{*1*}**	**2{*3*}**	5	0	**3{*1*,*3*}**	0		
**VI**	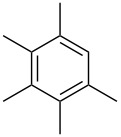 **1{*1*}**	**2{*1*}**	5	51 (51)^c^	**3{*1*,*1*}**	51		
**VI**	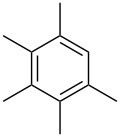 **1{*1*}**	**2{*1*}**	48	15 (15)^d^	**3{*1*,*1*}**	15		

^a^Reaction conditions: 1 equiv arene, 1 equiv alkyne, 1 equiv tetrafluoroboric acid, 0.1 mol % Au, 0.1 or 0.3 mol % AgBF_4_, 1,2-dichloroethane, 25 °C. ^b^The yield was determined by ^1^H NMR spectroscopy. ^c^Reaction performed with trifluoroacetic acid. ^d^Reaction performed without acid.

The catalytic activity of the complexes remained high also with less substituted substrates, complex **VI** being systematically superior to complex **VII**. The selectivity of the reaction was, however, hampered by the formation of significant amounts of products deriving from the addition of two molecules of alkyne to the arene (product type **4**), which was invariably recorded when more than one C–H group was available for reaction. Other by-products that were observed on using Pd catalysis, such as, e.g., products of double-bond isomerisation or deriving from insertion of more than one alkyne molecule into the same arene C–H bond, were however never detected with Au catalysts. Only in the case of *p*-xylene was the reaction again fully selective, albeit sluggish. Variations of the alkyne substrate made it apparent that electron-rich alkynes, such as 1-hexyne, are not viable substrates for this reaction, and that electron-poor, internal alkynes react only scarcely under these conditions.

**Scheme 3:**
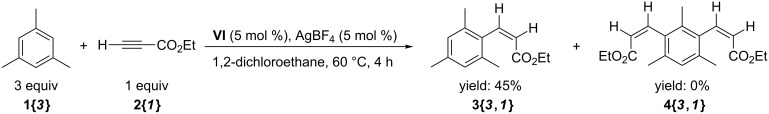
Hydroarylation experiment with catalyst **VI** under neutral conditions.

The high catalytic activity of complex **VI** prompted us to evaluate its efficiency also under less acidic conditions. Hydroarylation of ethyl propiolate with pentamethylbenzene run with 0.1% **VI** and 0.1% AgTFA in HTFA yielded 51% pure monohydroarylated product after 5 h. On the other hand, the reaction run with 0.1% **VI** and 0.1% AgBF_4_ under neutral conditions yielded only 15% product after 48 h. Thus, the nature and amount of acid have a very strong influence on catalytic efficiency, as in the case of catalysis by palladium(II) NHC complexes [13]; in contrast to Pd, though the catalyst remains slightly active even under neutral conditions. This result was expected, as in early examples of the use of gold catalysts for intermolecular alkyne hydroarylations the reaction was invariably done without acid addition, although much more forceful conditions (larger amount of catalyst, higher temperature, longer reaction times) were applied [18–19,47]. In order to have a closer comparison between the catalytic efficiency of **VI** and that of the previously employed gold(I) catalysts, such as AuCl(PPh_3_), we subjected complex **VI** to the same catalytic test performed by Reetz and Sommer with the phosphino complex (Scheme 3) [18]. The catalytic test performed with catalyst **VI** resulted in the exclusive formation of the hydroarylation product in 45% yield. Catalyst AuCl(PPh_3_) was reported instead to produce, under the same reaction conditions, the hydroarylation product in 56% yield, together with 28% yield of the product deriving from insertion of two alkyne molecules into two C–H bonds of mesitylene [18]. Thus, it can be stated that catalyst **VI** is apparently less active but more selective under these neutral reaction conditions.

**Scheme 4:**
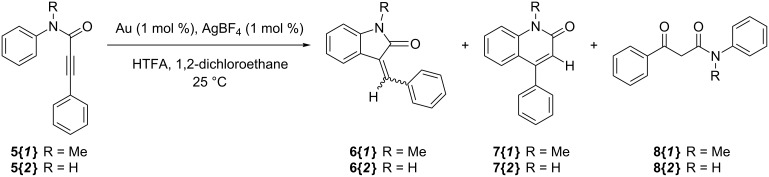
Intramolecular cyclisation through hydroarylation investigated in this work.

Finally, we preliminarily investigated the capability of complexes **VI** and **VII** to act as catalysts for intramolecular alkyne hydroarylation reactions. As mentioned in the Introduction, gold salts and complexes have been extensively employed for intramolecular cyclisations of this kind [35–46], but in most instances only terminal pendant alkynes have been employed as reacting groups. Furthermore, to the best of our knowledge substrates with amido tethers between the aryl and the alkyne, such as substrates **5** (Scheme 4), have never been reported to undergo cyclisation with gold catalysts, whereas there are examples of the use of palladium catalysts with these substrates leading to different products in dependence on the reaction conditions: reaction under neutral conditions produces the 5-*exo*-*dig* cyclisation product **6** [59], whereas in the presence of an acid the 6-*endo*-*dig* product **7** is formed [60]. Thus, we set out to evaluate the reactivity of substrates **5** with catalysts **VI** and **VII**.

Under neutral conditions the reaction gave no yield in the desired cyclised product and the substrate was recovered unchanged in all cases. Therefore, we moved to investigate the reaction in the presence of trifluoroacetic acid, using a reaction protocol previously employed by Fujiwara for running analogous reactions with palladium(II) catalysts [60]. The results are reported in Table 2.

**Table 2:** Intramolecular alkyne hydroarylation under acidic conditions.^a^

Substrate	Catalyst	Conversion (%)^b^	Yield (%)^b^			

**5{*1*}**	**VI**	89	**7{*1*}**	12	**8{*1*}**	78
**5{*1*}**	**VII**	31	**7{*1*}**	10	**8{*1*}**	-
**5{*2*}**	**VI**	54	**7{*2*}**	7	**8{*2*}**	47
**5{*2*}**	**VII**	0	**7{*2*}**	0	**8{*2*}**	0

^a^Reaction conditions: 1 equiv substrate, 20 equiv trifluoroacetic acid, 1 mol % Au, 1 mol % AgBF_4_, 1,2-dichloroethane, room temperature, 24 h. ^b^The conversion and the yields were determined by ^1^H NMR spectroscopy.

Catalyst **VII** was completely inactive even under acidic conditions with substrate **5{*2*}**, whereas with substrate **5{*1*}** it reacted sluggishly forming complex product mixtures containing also the 6-*endo*-*dig* cyclisation product **7{*2*}**. On the other hand, with complex **VI** moderate to very good conversions of the substrates were obtained, but the main reaction product was invariably the product of hydration of the triple bond **8**, whereas the 6-*endo*-*dig* cyclisation product **7** was present in minor amounts. Gold(I) NHC complexes such as **VI** are known to be extremely efficient catalysts for alkyne hydration [61], hence it can be expected that alkyne hydration by traces of water may become a serious competitive reaction despite the low concentration of water in the reaction mixture. On the basis of the above, it can be hypothesised that in order to steer the reaction towards the hydroarylation product, more activating, hence electron-donating substituents should be installed on the aryl ring. Experiments towards this goal are currently underway.

## Conclusion

In conclusion, we have demonstrated that gold complexes with N-heterocyclic carbenes are active catalysts for alkyne hydroarylations under acidic conditions. Mononuclear complexes appear more active than dinuclear ones, and gold(I) complexes are more active and selective than analogous gold(III) complexes. Under neutral reaction conditions, mononuclear gold(I) NHC complexes appear less active but more selective than the corresponding triphenylphosphine complexes. Finally, tests performed on the intramolecular hydroarylation of substrates **5{*1*}** and **5{*2*}** indicate that the reaction does not take place under neutral conditions, whereas under acidic conditions products of alkyne hydration by traces of water present in the reaction mixture are mainly formed, together with low yields of the 6-*endo*-*dig* cyclisation product. Possibly the installation of an electron-donating group on the aryl ring will improve the efficiency of the hydroarylation process.

## Experimental

All manipulations were carried out using standard Schlenk techniques under an atmosphere of dry argon or dinitrogen. The reagents were purchased at Sigma–Aldrich or Merck as high-purity products and generally used as received. All solvents were dried by standard procedures and distilled under dinitrogen prior to use. Complexes **I** [55], **II**–**V** [54] and **VII** [58], as well as substrates **5{*1*}** and **5{*2*}** [59] were prepared according to literature procedures. NMR spectra were recorded on a Bruker Avance 300 MHz (300.1 MHz for ^1^H and 75.5 MHz for ^13^C); chemical shifts (δ) are reported in units of parts per million (ppm) relative to the residual solvent signals.

**Catalytic tests.** General procedure for the intermolecular hydroarylation: In a 100 mL three-necked, round-bottomed flask were placed the arene (13.2 mmol), the Au complex (0.013 mmol for mononuclear complexes, 0.0065 mmol for dinuclear complexes) and AgBF_4_ (0.013 mmol to 0.052 mmol, depending on the Au complex employed). The flask was evacuated and filled with argon, after which the acid (13.2 mmol) and 1,2-dichloroethane (the quantity necessary to reach a total volume of 6.3 mL) were added. Finally, the alkyne (13.2 mmol) was introduced, and the flask was placed in a water bath thermostated at 25 °C and vigorously stirred. Aliquots of the reaction mixture (around 0.2 mL) were periodically withdrawn from the reactor and analysed by ^1^H NMR.

General procedure for the intramolecular hydroarylation: In a Schlenck tube were placed the substrate (1.00 mmol), the Au complex (0.010 mmol) and AgBF_4_ (0.010 mmol). The flask was evacuated and filled with argon, after which 1,2-dichloroethane (2 mL) and trifluoroacetic acid (1.5 mL, 20 mmol) were added. The resulting mixture was vigorously stirred at room temperature for 24 h. The reaction mixture was subsequently poured into a saturated aqueous NaCl solution (20 mL) and neutralized with a saturated aqueous NaHCO_3_ solution. The residual substrate and products were extracted into diethyl ether (20 mL). The resulting ethereal solution was washed with a saturated aqueous NaCl solution (10 mL) and water (10 mL), dried over Na_2_SO_4_ and evaporated to dryness. The residue was analysed by ^1^H NMR.
